# German second-opinion network for testicular cancer: Sealing the leaky pipe between evidence and clinical practice

**DOI:** 10.3892/or.2014.3153

**Published:** 2014-04-24

**Authors:** FRIEDEMANN ZENGERLING, MICHAEL HARTMANN, AXEL HEIDENREICH, SUSANNE KREGE, PETER ALBERS, ALEXANDER KARL, LOTHAR WEISSBACH, WALTER WAGNER, JENS BEDKE, MARGITTA RETZ, HANS U. SCHMELZ, SABINE KLIESCH, MARKUS KUCZYK, EVA WINTER, TOBIAS POTTEK, KLAUS-PETER DIECKMANN, ANDRES JAN SCHRADER, MARK SCHRADER

**Affiliations:** 1 Department of Urology, University Hospital of Ulm, Ulm, Germany; 2 Department of Urology, University Hospital Hamburg-Eppendorf, Hamburg, Germany; 3 Department of Urology, RWTH Aachen University, Aachen, Germany; 4 Department of Urology, Alexianer Krefeld GmbH, Krefeld, Germany; 5 Department of Urology, University of Düsseldorf, Düsseldorf, Germany; 6 Department of Urology, University of Munich, Munich, Germany; 7 Men’s Health Foundation, Berlin, Germany; 8 Department of Urology, Federal Armed Forces Hospital, Hamburg, Germany; 9 Department of Urology, University Hospital of Tübingen, Tübingen, Germany; 10 Department of Urology, Medical Center Rechts Der Isar, Technical University of Munich, Munich, Germany; 11 Department of Urology, Federal Armed Forces Hospital, Koblenz, Germany; 12 Center of Reproductive Medicine and Andrology-Clinical Andrology, University Hospital of Münster, Münster, Germany; 13 Department of Urology, Hannover Medical School, Hannover, Germany; 14 Department of Urology, HELIOS Hospital Schwerin, Schwerin, Germany; 15 Department of Urology, Asklepios Westklinikum, Hamburg, Germany; 16 Department of Urology, Albertinen Hospital, Hamburg, Germany

**Keywords:** second-opinion network, testicular cancer, quality of care, treatment scope

## Abstract

In 2006, the German Testicular Cancer Study Group initiated an extensive evidence-based national second-opinion network to improve the care of testicular cancer patients. The primary aims were to reflect the current state of testicular cancer treatment in Germany and to analyze the project’s effect on the quality of care delivered to testicular cancer patients. A freely available internet-based platform was developed for the exchange of data between the urologists seeking advice and the 31 second-opinion givers. After providing all data relevant to the primary treatment decision, urologists received a second opinion on their therapy plan within <48 h. Endpoints were congruence between the first and second opinion, conformity of applied therapy with the corresponding recommendation and progression-free survival rate of the introduced patients. Significance was determined by two-sided Pearson’s χ^2^ test. A total of 1,284 second-opinion requests were submitted from November 2006 to October 2011, and 926 of these cases were eligible for further analysis. A discrepancy was found between first and second opinion in 39.5% of the cases. Discrepant second opinions led to less extensive treatment in 28.1% and to more extensive treatment in 15.6%. Patients treated within the framework of the second-opinion project had an overall 2-year progression-free survival rate of 90.4%. Approximately every 6th second opinion led to a relevant change in therapy. Despite the lack of financial incentives, data from every 8th testicular cancer patient in Germany were submitted to second-opinion centers. Second-opinion centers can help to improve the implementation of evidence into clinical practice.

## Introduction

Implementing scientific evidence into clinical practice requires an active translational process consisting of several consecutive elements, the so-called ‘research-to-practice pipeline’ (1). The quality of the pipeline will determine the size of any information leak. A considerable number of studies emphasize the often inadequate translation of evidence-based guidelines into clinical practice (2,3).

The aim of the ‘National Second-Opinion Project on Testicular Cancer’ initiated in Germany in 2006 was to optimize the flow of evidence into clinical practice by sealing the leaky pipeline. Another aim was to maximize the availability of cutting-edge clinical knowledge regarding testicular cancer management by establishing a nationwide second-opinion network run by 31 selected colleagues acting as second-opinion givers. Short reaction times and immediate incorporation of new research results were intended to increase the pipeline flow rate and overcome barriers to translating evidence into clinical practice (4,5).

In a previous interim analysis of 642 cases, we demonstrated the high demand for second opinions before primary therapy in Germany (6). In the present study, we report the results of the ‘National Second-Opinion Project on Testicular Cancer’ after a period of 5 years, including data from the first 2 years of follow-up. The project is based on the hypothesis that patients benefit from second opinions offered systematically before the initiation of treatment after orchiectomy.

## Materials and methods

In 2006, a modular web-based interactive database program was developed by the German Testicular Cancer Study Group (GTCSG) in cooperation with the DOCxcellence Co., Berlin, Germany. The system was examined by the Data Protection Commissioner of the State of Berlin and found to be in accordance with the law. It also received security clearance for nationwide application. Patients must provide their informed consent to their personal data being recorded and used by the system.

**Figure 1:**
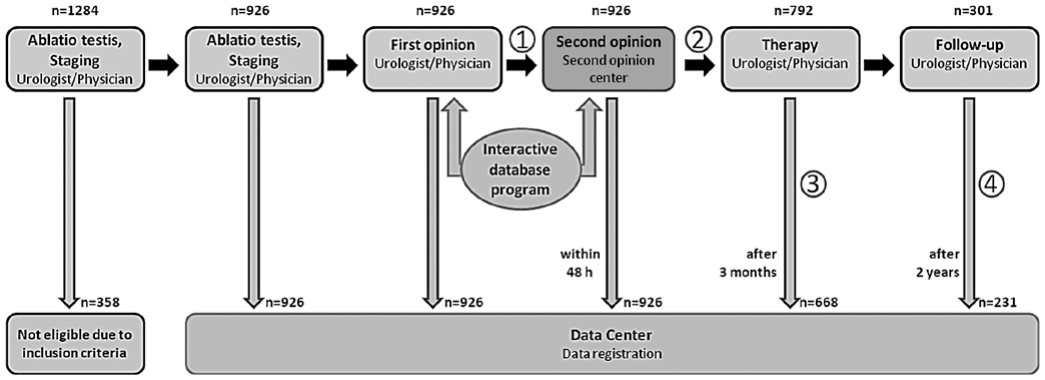
Concept and data flow of the second-opinion project for patients with testicular cancer. After one-time-only user registration, any urologist is able to request a second opinion from one of the 31 participating second-opinion centers. The primary clinical, pathological and imaging data of the respective patient can be put in a 21-item data mask online (step 1). A system-immanent algorithm helps to avoid misinformation. Physicians at the respective second opinion center are then required to recommend a therapy (step 2). In complex cases, they can enter into a dialogue with the physician making the inquiry. The data center registers the applied therapy 3 months after the request for a second opinion (step 3) and carries out a follow-up 2 years later (step 4).

The system is available to all urologists in Germany free of charge, regardless of whether they work in clinical departments or private practices (http://www.zm-hodentumor.de). It provides a second opinion for therapy planning after the primary diagnosis and staging of germ cell cancer. The system functions as recently described (Fig. 1) (6). During the study period, 31 clinical departments functioned as second-opinion centers in Germany and Austria (7). Multidimensional criteria were used to appoint them. The main selection criterion was an active role in developing the treatment guidelines for germ cell cancer (8,9). Centers were also required to provide proof of clinical and research activities in the field of germ cell cancer.

The first endpoint of the present investigation was the rate of discordance between the first and second opinion (i.e., between the therapy planned by the inquiring urologist and that recommended by the second-opinion giver). Another endpoint was the degree of compliance with the recommended treatment. A third endpoint was the treatment outcome as measured by the progression-free survival (PFS) rate.

### Statistical analysis

For this interim analysis, the anonymized patient data were evaluated with SPSS v.11.0 by the Dross Institute of Statistics at the Free University of Berlin. The advice seekers may choose 1 of 15 different treatment options as their first opinion, including ‘Findings are not conclusive enough for a definite recommendation’ and ‘Others: free text.’

The second opinions were multiple-choice answers relating to the same 15 categories. Second-opinion centers may then recommend up to four alternative treatment options and make free-text comments. The initially planned treatment was considered to be concordant with the second opinion if it coincided with at least one recommendation made by the second-opinion center. Discordant cases were further differentiated as follows: i) first opinion favors more extensive treatment, i.e. ‘overtreatment’ according to guidelines; ii) first opinion favors less extensive treatment, i.e. ‘undertreatment’ according to guidelines; iii) discordance with no substantial difference in the scope of therapy.

In these cases, the treatment scope of alternative options was assessed in relation to the clinical tumor stage for which it was recommended by the guidelines (8,9). Significance was determined by Pearson’s χ^2^ test (two-sided). Standardized residuals for cells in the cross-classification table were used for content-related interpretation of a significant result. Significance was also determined using a Monte Carlo simulation with 10,000 trials and a 99% confidence interval.

## Results

A total of 1,284 requests were submitted by 350 urologists/physicians to the 31 second-opinion centers from November 2006 to October 2011. The present interim analysis only took into account the requests from colleagues working in private practice or hospital departments but not at one of the 31 institutions serving as second-opinion centers; the latter were excluded as unsuitable for assessing the additional benefit of a second-opinion network, since they would have had access to an expert opinion anyway. At the time of the interim analysis, 926 cases met this inclusion criterion and were thus eligible. Patient characteristics are given in Table I.

The 3-month follow-up deadline was reached in 792 cases at the time of the analysis. The applied therapy may be ascertained in 668/792 cases, which corresponds to a response rate of 84.3%. The 2-year follow-up has thus far been completed in 301 patients, and aftercare information was provided for 236 of them, corresponding to a response rate of 78.4%.

The first-opinion therapy suggested by the advice seeker was concordant with the second-opinion therapy recommended by the centers in only 58.0% of the cases. Discordance between first and second opinions was found in 39.5%, while deviation from recommendations remained unclear in the remaining cases (2.5%). The discordance rate increased significantly with tumor stage (Pearson’s χ^2^ test; <0.001; Table II), the difference being most obvious between stages I and IIa/IIb. Discordance was not associated with histology (seminomas and non-seminomas; Pearson’s χ^2^ test, p=0.442).

In the case of a discordant second opinion, the scope of the first-opinion therapy was assessed to determine whether it involved over- or undertreatment. The second-opinion treatment was less extensive in 28.1% and more extensive in 15.6% of the discordant cases than that originally planned. In another 56.3% of the cases, there was no substantial difference in the scope of the treatment between first and second-opinion.

In 30.8% of the cases (206/668), the applied therapy was not in accordance with either the first or the second opinion. The remaining 462 cases showed a clear tendency towards compliance with the second rather than the first opinion (85.3 vs. 14.7%).

Two-year PFS data were available for 188 patients at the time of the interim analysis. A total of 18 relapses or progressive tumors were reported. This corresponded to a 2-year PFS of 90.4% for the total patient population. The 2-year PFS stratified by tumor stage was 95.2% (118/124) for stage I, 92.0% (23/25) for stage IIa–IIb and 75% (24/32) for stage ≥IIc (Table III). Relapse or progression of the tumor was diagnosed by routine imaging procedures in 15/18 cases (83.3%), by biopsy (with previous imaging) in 6/18 cases (33.3%) and by elevated tumor markers in only 4/18 cases (22.2%).

## Discussion

A multidisciplinary set of quality indicators has recently been developed for the treatment of testicular cancer patients (10). Here, the concept of treatment quality is extended to include indicators beyond survival. The incorporation of new research results and guideline conformity play a crucial role in this context. The question as to what procedure conforms to the guidelines can be scrutinized systematically or even individually in cases where doubts arise regarding treatment decisions. The problem is that doubts do not always arise when they should, and then the inappropriate approach is taken with firm conviction. This suggests that guideline conformity should be systematically checked by a second-opinion system, since the research- or guideline-to-practice pipeline has often proved to be leaky in the past (2,3).

The establishment of the national second-opinion network for testicular germ cell cancer by the German Testicular Cancer Study Group (GTCSG) in 2006 (6) represented a worldwide unique approach for actively propagating and implementing guideline recommendations. Given the decentralized health care system in Germany with treatment of only a very small average annual number of testicular tumors per institution, this network was intended to promote the delivery of cutting-edge therapy to a large number of testicular cancer patients.

In 2011, approximately every 8th newly diagnosed case of testicular cancer in Germany received a second opinion from our service (n=465/3,950; 11.8%) (11). Given the segmental structure of the German health care system, which is reflected in many countries worldwide, this was a substantial quota. It suggests that physicians do actually appreciate being able to request a second opinion, provided that: (i) it does not take much time, and (ii) it does not involve referring the patient to another physician.

This high acceptance rate demonstrates the high level of responsibility taken by the participating advice seekers, since there were no financial incentives for the additional time and effort. They were only committed to ensuring that their patients received the best primary therapy available. The same was true for the participating physicians in the second-opinion centers, who provided their second opinion very quickly (median reaction time of only 10.64±16.99 h) and free of charge.

A 2006 study showed that publication of the GTCSG guidelines significantly influenced the management of testicular cancer patients at a university treatment center in 1999 (12). Our data reveal a 40% discrepancy between the first and second opinion, which probably reflects an up to 40% deviation of primary caregivers from guideline recommendations. Furthermore, patterns of care studies have shown that compliance with recommended imaging procedures decreases during follow-up in testicular cancer patients (13).

Of note, nearly one third of the applied therapies were not in accordance with either the second opinion or the initially planned therapy. Limited implementation definitely accounts for an attenuated effect of the second opinions. However, imprecise data input of the second-opinion seekers due to the relatively long 3-month interval between the initiation of treatment and its registration by our data center may have also played a role in the relatively high rate of ‘third opinions’. Another factor may be the often suboptimal adherence to medical advice among testicular cancer patients (14).

Today, testicular cancer patients have an overall cure rate of more than 95% (11). For this reason and owing to the comparatively young age of onset, considerable attention is paid to treatment-related morbidity in these patients. In contrast to other malignancies, the relapse rate here is not indicative of treatment quality, since overtreatment, for example, may increase toxicity and long-term morbidity without affecting the relapse rate.

Thus, the aim of treatment is, on the one hand, not to jeopardize the excellent cure rate of the disease by undertreatment, and, on the other hand, to avoid the morbidity associated with overtreatment. The second-opinion project is committed to achieving this goal.

Approximately every 6th second opinion (n=160/926; 17.3%) prompted a relevant change of therapy, which confirms the findings of our first interim report (6). Thus, with reference to all cases submitted, over- or undertreatment is avoided in 80 German patients per year by following the second-opinion recommendations. Another 103 patients per year receive optimized treatment due to minor changes if adjusted therapeutic strategies with no relevant changes in scope are also taken into account.

The current analysis provides first follow-up data on the testicular cancer patients treated in the second-opinion network; 18 of 188 patients (9.6%) had relapse or progression of the disease. Stratified by both the total patient population and the tumor stage, the 2-year PFS did not differ from that of patients receiving second-opinion treatment in clinical trials (15,16). A correlation of the clinical relapse rate with the second-opinion conformity of the applied therapy will be validated in a future analysis as soon as more 2-year follow-up data are available.

Our results are limited by some conceptual shortcomings in the present second-opinion project. For example, the relatively high number of second-opinion givers may raise concerns about second-opinion quality being diluted by too many participating physicians. The basic idea was to distribute second-opinion requests evenly among the consultants in order to guarantee a short reaction time. However, this goal was not achieved: the distribution turned out to be relatively uneven (n=0 to n=199; Table I). Indeed, only 21 of 31 second-opinion givers had more than one second-opinion request to answer during the study period. It thus seems expedient to rethink the procedure for selecting second-opinion givers in the future.

Exchange with experts from other fields (e.g. radiation oncology, medical oncology, nuclear medicine) was maintained by the second-opinion giver dependent on each individual case, but not carried out systematically. The latter would have been desirable in the decision-making process, reflecting another weakness of the project.

Two-year PFS was used to measure treatment quality. We are aware that, in the face of a changing treatment landscape with a trend toward avoiding late toxicities of cancer therapy, this parameter does not cover all the most important aspects of cutting-edge testicular cancer treatment. Unfortunately, therapy-associated morbidities were beyond the scope of our project, and the follow-up period is still too short to give them adequate consideration.

Nevertheless, an unexpected finding is the high level of project participation in the strongly finance-oriented German health care system. The ideal aim of making optimal treatment decisions by discussing cases with specialized centers can be achieved in a nearly conflict-free fashion by the established system. The physicians seeking advice did not run the risk of being discredited or losing their patients by referral to (more) qualified colleagues. Moreover, the time required was so small that it posed no obstacle. All this helps to explain the large number of highly-motivated project participants.

In conclusion, the ‘National Second-Opinion Project for Testicular Cancer’ demonstrates for the first time a new way to improve the ‘research-to-practice pipeline’. The possibility of nearly obstacle-free online communication among physicians with no financial disadvantage or loss of authority is deemed to have improved the quality of care delivered to testicular cancer patients in Germany. The ‘National Second-Opinion Project for Testicular Cancer’ should therefore serve as a model for establishing further second-opinion networks for other health care systems or diseases.

## Figures and Tables

**Table I:** Patient characteristics.

Variables	n
Total second-opinion requests	1,284
Requests from colleagues in private practice and hospital departments	926^a^
Answers/second-opinion giver (range, 0–199)	29.9±51.1
Average patient age in years	37.0±11.3
Histological finding at time of diagnosis of germ cell tumor	
Seminoma	424 (45.8%)
Average age in years	40.9±10.7
Non-seminoma	454 (49.0%)
Average age in years	31.8±11.3
Unclear whether seminoma or non-seminoma	48 (5.2%)

a 358 second-opinion requests from colleagues working at one of the 31 institutions serving as second-opinion centers were excluded from the present analysis.

**Table II:** Concordance between the first and second opinion in relation to tumor stage (n=926).

	First and second opinion					
		
Clinical tumor stage(categorized)	Concordant n (%)	Discordant: second opinion more extensive therapy n (%)	Discordant: no clear difference in the scope of therapy n (%)	Discordant: first opinion more extensive therapy n (%)	Concordance status not clear n (%)	Total n (%)
I	357 (66.0)	26 (4.8)	87 (16.1)	65 (12.0)	6 (1.1)	541 (100)
IIa, IIb	78 (44.3)	15 (8.0)	55 (31.3)	21 (11.9)	8 (4.5)	177 (100)
IIc, IIIa, IIIb, IIIc^a^	83 (48.8)	15 (8.8)	49 (28.8)	16 (9.4)	7 (4.1)	170 (100)
Unknown	19 (48.7)	1 (2.6)	15 (41.0)	1 (2.6)	2 (5.1)	38 (100)
Total	537 (58.0)	57 (6.2)	206 (22.2)	103 (11.1)	23 (2.5)	926 (100)

Patients were classified according to the Lugano classification. The scope of therapy was evaluated according to the guideline recommendations for the respective tumor stage (7,8). Pearson’s χ^2^ (two-sided) showed that the percentage of discrepant recommendations increased significantly with increasing tumor stage (p<0.001).

a 52.5% good prognosis group; 29.0% intermediate prognosis group; 18.5% poor prognosis group.

**Table III:** Two-year progression-free survival in relation to tumor stage (n=188).

Clinical tumor stage (categorized)	Progression-free at 2-year follow-up		Total n (%)

	Yes n (%)	No n (%)	
Ia, Ib, Is	118 (95.2)	6 (4.8)	124 (100)
IIa, IIb	23 (92.0)	2 (8.0)	25 (100)
IIc, IIa, IIIb, IIIc	24 (75.0)	8 (25.0)	32 (100)
Unknown	5 (71.4)	2 (28.6)	7 (100)
Total	170 (90.4)	18 (9.6)	188^a^ (100)

Patients were classified according to the Lugano classification. The progression-free survival rate was calculated using the 2-year follow-up data.

a Analysis excluded 48 of 236 cases as the reply was ‘unknown’.

## References

[1] Glasziou P, Haynes B (2005). The paths from research to improved health outcomes. Evid Based Nurs.

[2] Rawlins MD (2004). NICE work - providing guidance to the British National Health Service. N Engl J Med.

[3] Raine R, Sanderson C, Black N (2005). Developing clinical guidelines: a challenge to current methods. BMJ.

[4] McNeil BJ (2001). Shattuck Lecture - Hidden barriers to improvement in the quality of care. N Engl J Med.

[5] Julian DG (2004). Translation of clinical trials into clinical practice. J Intern Med.

[6] Schrader M, Weissbach L, Hartmann M (2010). Burden or relief: do second-opinion centers influence the quality of care delivered to patients with testicular germ cell cancer?. Eur Urol.

[7] ZMZ-Ärzte-Projekt Zweitmeinung Hodentumor.

[8] Krege S, Beyer J, Souchon R (2008). European consensus conference on diagnosis and treatment of germ cell cancer: a report of the second meeting of the European Germ Cell Cancer Consensus group (EGCCCG): part I. Eur Urol.

[9] Krege S, Beyer J, Souchon R (2008). European consensus conference on diagnosis and treatment of germ cell cancer: a report of the second meeting of the European Germ Cell Cancer Consensus Group (EGCCCG): part II. Eur Urol.

[10] Vlayen J, Vrijens F, Devriese S, Beirens K, Van Eycken E, Stordeur S (2012). Quality indicators for testicular cancer: a population-based study. Eur J Cancer.

[11] (2012). Robert Koch-Institut und die Gesellschaft der epidemiologischen Krebsregister in Deutschland e.V. (Hrsg). Krebs in Deutschland 2007/2008. 8. Ausgabe. Journal.

[12] Schrader AJ, Ohlmann CH, Rossmanith S, Hofmann R, Heidenreich A (2006). Impact of evidence-based interdisciplinary guidelines on testis cancer management. Cancer.

[13] Yu HY, Madison RA, Setodji CM, Saigal CS (2009). Quality of surveillance for stage I testis cancer in the community. J Clin Oncol.

[14] Moynihan C, Norman AR, Barbachano Y (2009). Prospective study of factors predicting adherence to medical advice in men with testicular cancer. J Clin Oncol.

[15] Berrino F, De Angelis R, Sant M (2007). Survival for eight major cancers and all cancers combined for European adults diagnosed in 1995–99 results of the EUROCARE-4 study. Lancet Oncol.

[16] Rosen A, Jayram G, Drazer M, Eggener SE (2011). Global trends in testicular cancer incidence and mortality. Eur Urol.

